# Nanoscale metal–organic frameworks coated with poly(vinyl alcohol) for ratiometric peroxynitrite sensing through FRET†
†Electronic supplementary information (ESI) available: Details about the synthesis and characterization of NMOF ONOO^–^ sensors. See DOI: 10.1039/c7sc01077j



**DOI:** 10.1039/c7sc01077j

**Published:** 2017-05-18

**Authors:** Zhaoyang Ding, Jinyun Tan, Gang Feng, Zhen Yuan, Changfeng Wu, Xuanjun Zhang

**Affiliations:** a Faculty of Health Sciences , University of Macau , Macau SAR , China . Email: xuanjunzhang@umac.mo; b Department of Biomedical Engineering , Southern University of Science and Technology , Shenzhen , Guangdong 518055 , China

## Abstract

This work describes a facile yet powerful approach to energy-transfer NMOF (nanoscale metal–organic framework) fabrication for ratiometric peroxynitrite (ONOO^–^) sensing. Poly(vinyl alcohol) (PVA) is chosen to organize the energy donor (NMOF) and acceptor (molecular probes). PVA can conveniently graft onto the NMOF surface and bind to the molecular probes bearing the arylboronic acid group through multiple weak coordination interactions. Due to efficient Förster resonance energy transfer (FRET), the bright blue fluorescence of the NMOF is quenched while the green or red emission from the acceptor is enhanced. Upon reacting with ONOO^–^, the ONOO^–^ sensors depart from the NMOF and the FRET is interrupted and the fluorescence of the NMOF recovered. Based on this strategy, we developed two ratiometric ONOO^–^ nanosensors for the detection of ONOO^–^ in solutions and living cells. This work is the first report of NMOF ONOO^–^ sensors through FRET and could inspire the design of other NMOF based chemical sensors and biosensors.

## Abstract 2

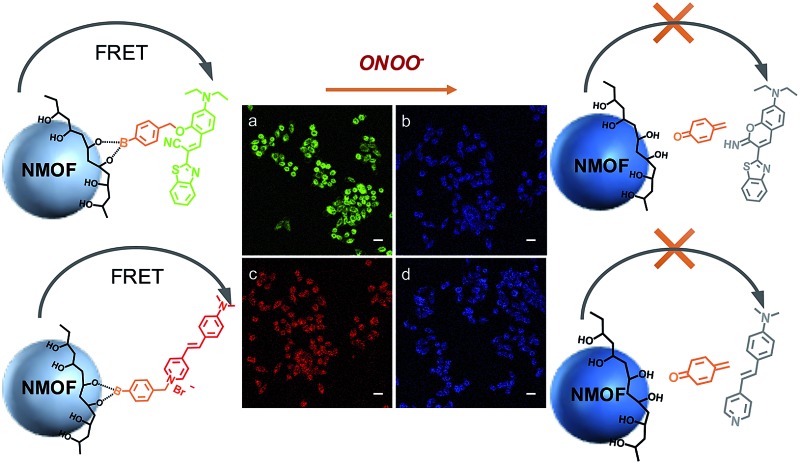
This work describes a facile yet powerful approach to energy-transfer NMOF (nanoscale metal–organic framework) fabrication for ratiometric peroxynitrite (ONOO^–^) sensing.

## Introduction

Metal–organic frameworks (MOFs) have emerged as a class of important materials in the past few decades for their diverse structures, tunable compositions and porosities, and unique properties.^1–5^ Recently, NMOFs have rapidly gained attention because of their potential applications including biomedical imaging,^6–8^ drug delivery,^9–12^ photodynamic therapy,^13^ chemical sensors,^14–20^ and so on. NMOFs possess potential advantages over existing nanoparticles in the design of light-harvesting systems for ratiometric sensing applications. Firstly, the densely packed oriented structures enable MOF materials to have a large absorption cross-section per unit volume to harvest light.^21–23^ Secondly, the molecular sensors (energy acceptors in this FRET system) can be encapsulated in pores, embedded into a MOF skeleton, or linked to a MOF surface by postsynthesis.^22,24–26^ Upon reacting with analytes, the energy transfer from the MOF to the molecular sensor switches from turn-off to turn-on, leading to a much more accurate ratiometric measurement compared to single molecular sensors.

Due to the aggregation effect of bare NMOFs, proper surface modification is needed to improve the water dispersibility or various further functionalization of NMOFs for biomedical applications. NMOFs can be coated with silica,^27^ peptides,^28,29^ proteins,^26,30^ polymers,^31^ *etc.* Although great progress has been made, functionalization on the surface of NMOFs and further conjugation with molecular probes is still very challenging. Here, we report a simple yet powerful surface coating for the construction of a fluorescent nanosensor for the ratiometric detection of peroxynitrite. Peroxynitrite (ONOO^–^) plays an important role in physiology and pathology as an extremely reactive oxygen species (ROS) in living systems. It is reported that ONOO^–^ has implications in cancer, diabetes, Alzheimer’s disease, arthritis, and other diseases.^32–34^ It could respond to electron-rich moieties as a potent oxidant, which causes damage to proteins, DNA and other biomolecules in living systems.^35,36^ Immunostaining of 3-nitrotyrosine was the traditional biological assay for ONOO^–^.^37^ It has restricted uses because it is incompatible with living systems, and cannot satisfy the research needs for tracking ONOO^–^ in living cells in real time. Because of the short half-time of ONOO^–^ (10–20 ms) in biological systems, direct and unambiguous detection methods in living cells could not be achieved using traditional analytical methods. Therefore, a highly sensitive and efficient method for the accurate and direct detection of ONOO^–^ is desirable. In the past decade, many ONOO^–^ fluorescence sensors have been developed based on various strategies that rely on active ketones,^38,39^ aromatic nitration,^40^ metal complexes,^41^ and so on.^42^ Fluorescent nanosensors could achieve long-term tracking and prevent the release of binding molecule probes from the nanosensors before they begin to detect in living cells, which is significant during ONOO^–^ detection.

**Scheme 1:**
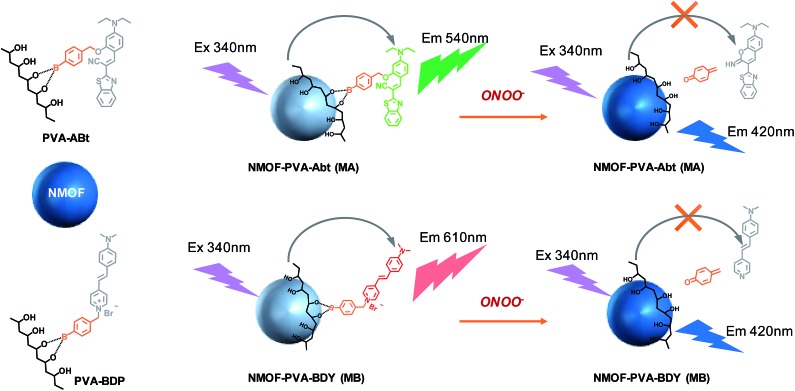
Schematic of the proposed sensing mechanism of NMOF sensors.

In this work, two ONOO^–^ sensors, ABt and BDP, are designed by taking advantage of the fast reaction of arylboronate with ONOO^–^. Poly(vinyl alcohol) (PVA) is chosen as an ideal agent for NMOF surface modification and conjugation with molecular probes. PVA has excellent biocompatibility and water solubility.^43^ The chelating moiety, –CHOH–CH_2_–CHOH–, of PVA can bind to the NMOF surface *via* multiple weak coordination with metal ions. This chelating moiety can also react with arylboronic acid to form a stable complex.^44^ As shown in Scheme 1, the molecular probes (ABt and BDP) containing arylboronic acid were designed to react with PVA. The conjugates, PVA–ABt and PVA–BDP, can be further coated on the surface of the NMOFs to form an energy-transfer nanosensor for the measurement of ONOO^–^.

Using the NMOF as the energy donor and the ONOO^–^ sensor as the energy acceptor, a FRET pair was built, which turned off the fluorescence of the NMOF *via* FRET. When ONOO^–^ is introduced, the ONOO^–^ sensor departs from the NMOF donor, leading to the interruption of the FRET process and therefore the turning on of fluorescence of the NMOF, accompanied by fluorescence quenching of the ONOO^–^ sensor. Based on the restored fluorescence of the NMOF and the quenched fluorescence of the ONOO^–^ sensors, sensitive and selective fluorescent sensors for the ratiometric determination of ONOO^–^ have been demonstrated.

## Results and discussion

### Synthesis and characterization of NMOF sensors

**Fig. 1:**
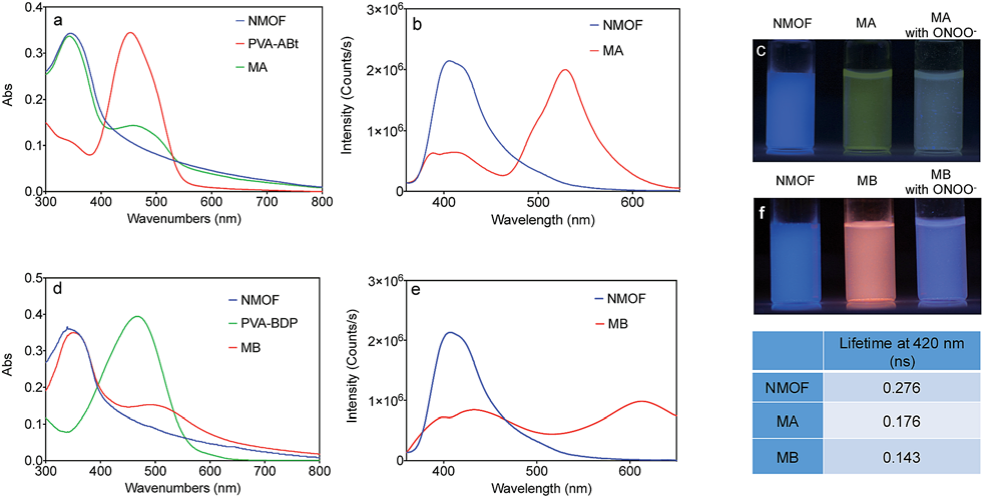
(a) Absorption spectra of the NMOF, PVA–ABt and MA; (b) fluorescence spectra of the NMOF and MA with an excitation wavelength at 340 nm; (c) naked eye photographs of the fluorescence change of the NMOF (10 mg L^–1^), MA (10 mg L^–1^) and MA (10 mg L^–1^) with 10.0 μM ONOO^–^. Excitation: 365 nm; (d) absorption spectra of the NMOF, PVA–BDP and MB; (e) fluorescence spectra of the NMOF and MB with an excitation wavelength at 340 nm; (f) naked eye photographs of the fluorescence change of the NMOF (10 mg L^–1^), MB (10 mg L^–1^) and MB (10 mg L^–1^) with 10.0 μM ONOO^–^. Excitation: 340 nm.

The ligand for the NMOF was synthesized by the successive Suzuki coupling method as in our previous work.^45^ It exhibits a very strong blue emission with a quantum yield of about 95% in DMF. The two carboxylic acid groups in the ligand can link to metal cations after deprotonation, which facilitates the formation of the MOF with Zr^4+^. As shown in Fig. 1, the optimal excitation and emission wavelengths of the NMOF were 340 nm and 420 nm, respectively.

Molecular probes bearing the arylboronate group, ABt and BDP (Scheme 1), were designed to detect ONOO^–^ and the synthetic routes are shown in Schemes S1 and S2.† Arylboronate could react with ONOO^–^ instantaneously to form hydroxy derivatives. ABt showed very weak fluorescence in dilute solution due to molecular twisting of the acrylonitrile moiety. BDP also showed very weak fluorescence in dilute solution, especially in polar solvents, due to strong intramolecular charge transfer (ICT). The product after reaction with ONOO^–^ showed blue-shifted fluorescence, which ensured a big difference in fluorescence upon detection. Meanwhile, the diethylamino group, which has a slight effect on strongly nucleophilic ONOO^–^, could decrease the electrophilicity of the boronate group to inhibit the nucleophilic attack of H_2_O_2_ to some extent. The fluorescence of ABt and BDP (which depart from the NMOF after detection) was negligible compared with the high-intensity fluorescence of the NMOF. In our nanosensor system, only a small amount of the molecular probes were linked onto the NMOF surface. Before reaction with the ONOO^–^ analyte, there was efficient energy transfer from the NMOF to the molecular probe. After the reaction, the molecular probe detached from the NMOF and energy transfer was switched off. These results indicate the recovery of NMOF fluorescence and the disappearance of molecular probe fluorescence.

PVA was introduced to this system as a linker between the NMOF and ONOO^–^ sensing molecules. As shown in Fig. 1a and d, the conjugates of PVA and the ONOO^–^ probes (PVA–ABt and PVA–BDP) show maximum absorption peaks at 455 nm and 475 nm, respectively. FT-IR spectra of PVA, PVA–ABt and PVA–BDP are shown in Fig. S4.† Compared with PVA, PVA–ABt has two characteristic peaks at 2210 cm^–1^ (the nitrile group from ABt) and 1348 cm^–1^ (the B–O group from ABt). PVA–BDP also has a peak at 1350 cm^–1^ (the B–O group from BDP). The results prove that ABt and BDP were connected to PVA. Fig. S5† presents the standard curves of PVA–ABt and PVA–BDP in various concentrations. After coating with PVA–ABt, the NMOF was washed with water and purified by centrifugation to remove any free PVA–ABt. The final nanosensor, MA, showed two peaks with the larger one at 340 nm and the smaller one at 455 nm. Fig. 1a indicates that the molecular sensor ABt was successfully linked to the MOF surface. Fig. 1d shows the absorbance spectra of the NMOF, PVA–BDP and MB. After coating with PVA–BDP, a small peak at 475 nm was also observed in the spectra of MB. The coating ratios (ABt or BDP to NMOF) were controlled to 2–4% by varying the reaction conditions and the amount of molecular probe added.

Fig. 1b and e show the fluorescence spectra of the NMOF, MA and MB using 340 nm excitation. The bare NMOF showed a sole emission band at 420 nm, while MA had two distinctive emission bands peaking at 420 nm and 530 nm, and MB had two distinctive emission bands peaking at 420 nm and 610 nm. The fluorescence at 530 nm and 610 nm was attributed to the emissions of ABt and BDP, respectively. The photoluminescence of ABt and BDP could not be efficiently excited with 340 nm light, hence the emissions at 530 nm or 610 nm were the result of FRET with energy transfer from the NMOF to the molecular probes (Scheme 1). As shown in Fig. S6,† the fluorescence spectra of the NMOF are markedly overlapped with the absorption spectra of PVA–ABt and PVA–BDP. Consequently, the NMOF could act as the energy donor and ABt or BDP as the energy acceptor. It is noted that ABt showed very weak fluorescence in dilute solution due to molecular twisting. However, grafting onto the MOF surface significantly restricted twisting. The FRET effect then further enhanced its fluorescence. BDP also showed very weak fluorescence in polar solvents such as water and alcohols due to the strong intramolecular charge transfer effect. Grafting onto the MOF surface or loading into the pores of the MOF could change the polarity of the local environment and enhance fluorescence, which was also observed in our previous study.^46^ The FRET effect of MA and MB can also be directly visualized by the naked eye in Fig. 1c and f. Under the light of a hand-held UV lamp at 365 nm, the NMOF exhibited blue fluorescence, while MA and MB exhibited green and red fluorescence respectively due to the FRET effect.

The fluorescence lifetimes were measured to study the energy transfer efficiency (*E* = (*τ*_D_ – *τ*_DA_)/*τ*_D_, where *τ*_D_ is the excited fluorescence lifetime (FL) of the donor) and the results are shown in the table inside Fig. 1. The NMOF, MA and MB were excited with 340 nm light and the lifetimes at 420 nm were measured. The obtained average lifetime of the NMOF was 0.276 ns, which decreased to 0.176 ns in MA and 0.143 ns in MB, respectively. The FRET efficiencies were calculated as 36.23% and 48.19% of MA and MB. The moderate energy transfer efficiencies are possibly due to the relatively longer distance between the inner parts of the MOF and the molecular probes.

**Fig. 2:**
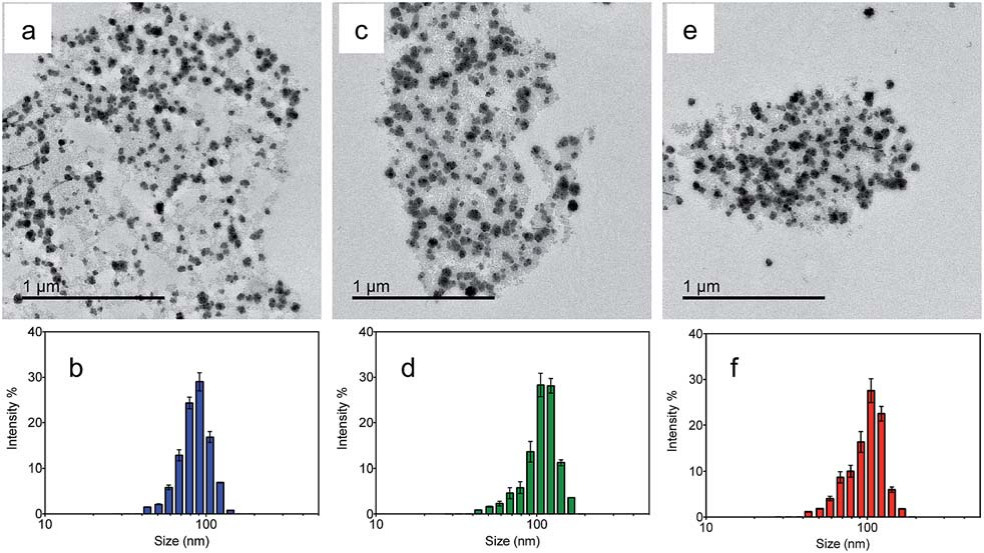
TEM images and DLS of the NMOF, MA and MB suspended in PBS buffer at pH 7.4. TEM images of the NMOF, MA and MB (a, c and e); diameter of the NMOF, MA and MB determined by DLS (b, d and f).

Transmission electron microscopy (TEM) was utilized to characterize the morphologies of the nanoparticles. Fig. 2a, c and e show the TEM images of the NMOF, MA and MB, respectively. The nanoparticles were well dispersed with diameters of 50–150 nm. The average hydrodynamic sizes dispersed in water were further measured by dynamic light scattering (DLS). The coating of the NMOF by PVA–ABt or PVA–BDP slightly increased the hydrodynamic sizes but no apparent aggregation was observed. As shown in Fig. 2b, d and f, the particles had hydrodynamic diameters of 86.80 nm (NMOF), 108.77 nm (MA) and 100.68 nm (MB), respectively. XRD and FT-IR analysis of the NMOF, MA and MB are shown in the ESI in Fig. S1 and S3.† The results show that after the coating experiments, the structures of the coated materials were not affected and were almost the same as before.

### Ratiometric fluorescence response of the nanosensors towards ONOO^–^

**Fig. 3:**
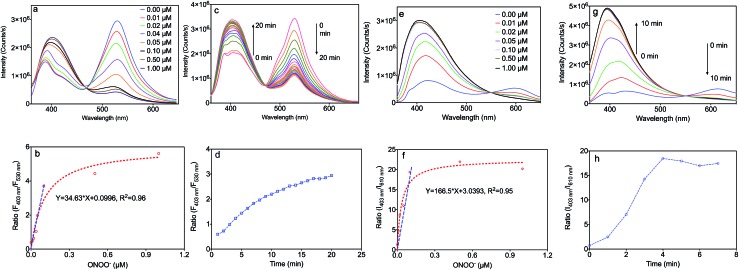
Properties of the MA and MB sensors. Excitation: 340 nm. (a) Fluorescence spectra of MA (1 mg L^–1^) upon addition of various amounts of ONOO^–^ (from 0.00 to 1.00 μM) in PBS buffer solution (pH 7.4) for 30 min; (b) non-linear correlation between the emission intensity ratio (*F*_403 nm_/*F*_530 nm_) and the concentration of ONOO^–^; (c) fluorescence spectra of MA (1 mg L^–1^) with the addition of 1.00 μM ONOO^–^ over time (0 to 20 min); (d) non-linear correlation between the emission intensity ratio (*F*_403 nm_/*F*_530 nm_) and time; (e) fluorescence spectra of MB (1 mg L^–1^) upon addition of various amounts of ONOO^–^ (from 0.00 to 1.00 μM) in PBS buffer solution (pH 7.4) for 30 min; (f) non-linear correlation between the emission intensity ratio (*F*_403 nm_/*F*_610 nm_) and the concentration of ONOO^–^; (g) fluorescence spectra of MB (1 mg L^–1^) with the addition of 1.00 μM ONOO^–^ over time (0 to 10 min); (h) non-linear correlation between the emission intensity ratio (*F*_403 nm_/*F*_610 nm_) and time.

The fluorescence responses of the MOF based nanosensors, MA and MB, were firstly studied in buffer solution at pH 7.4. Upon addition of ONOO^–^, these sensors exhibited a marked hypsochromic shift of the fluorescence spectra with isosbestic points at 465 nm (MA) and 555 nm (MB) (Fig. 3a and e). As shown in Fig. 3a, MA emitted green fluorescence with an emission maximum at 530 nm, which is a consequence of the extended π-conjugated system present in this sensor. Upon addition of ONOO^–^, the emission peak of MA at 530 nm decreased together with an increase of the blue band peak at 403 nm. Moreover, the ratio of the fluorescence intensities of MA at 403 nm to 530 nm in Fig. 3b linearly correlates with the concentration of ONOO^–^ from 0.0 to 0.1 μM, which indicates an accurate detection limit. The response time is also important during the detection of ONOO^–^, and the fluorescence spectra of MA reacting with ONOO^–^ over time, along with the correlation between the emission intensity ratio and time, are exhibited in Fig. 3c and d. The emission peak of MA at 530 nm decreased with the increase of the band at 403 nm over time from 0 to 20 minutes. The reaction came to equilibrium after 15 minutes, and we decided to choose 30 minutes in further experiments.

As shown in Fig. 3e, MB emits red fluorescence with an emission maximum at 610 nm, which is consistent with the fluorescence of the molecular probe, BDP. Upon addition of ONOO^–^, the emission peak of MB at 610 nm decreased together with an increase of the band at 403 nm corresponding to a blue colour. Moreover, the ratio of the fluorescence intensities of MB at 403 nm to 610 nm in Fig. 3f also linearly correlates with the concentration of ONOO^–^ from 0.0 to 0.1 μM. The fluorescence spectra of MB reacting with ONOO^–^ over time, along with the correlation between the emission intensity ratio and time, are shown in Fig. 3g and h. The emission peak of MA at 610 nm decreased with the increase of the band at 403 nm over time from 0 to 5 minutes. This reaction came to equilibrium after 5 minutes, and we decided to choose 10 minutes in further experiments.

**Fig. 4:**
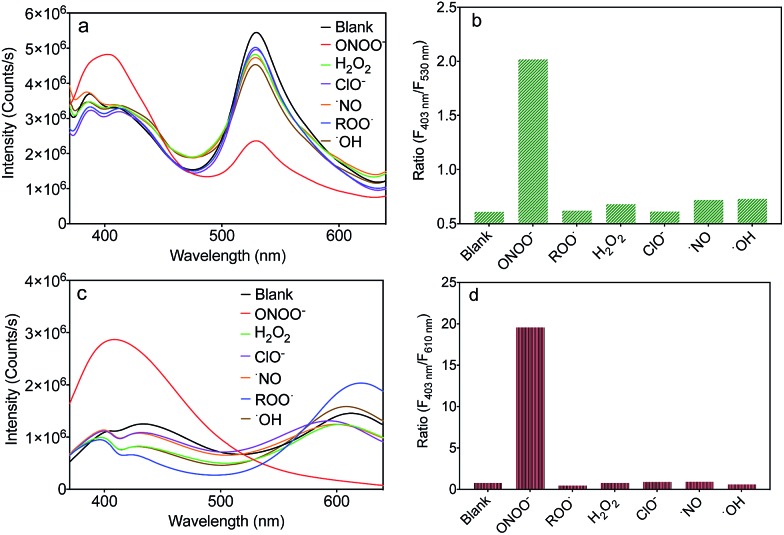
Fluorescence spectra of MA (a) and MB (c) in PBS buffer solution (pH 7.4) upon the addition of various ROS. Corresponding fluorescence intensity ratios of MA (*F*_403 nm_/*F*_530 nm_) (b) and MB (*F*_403 nm_/*F*_610 nm_) (d) in PBS buffer solution (pH 7.4) upon the addition of various ROS.

To evaluate the selectivity of MA and MB for ONOO^–^, the fluorescence spectra of MA and MB before and after the addition of common ROS, including H_2_O_2_, OCl^–^, ˙NO, ROO˙ and ˙OH, were recorded. As shown in Fig. 4a and c, only ONOO^–^ caused an obvious ratiometric fluorescent response in MA and MB. Furthermore, the other ROS did not cause observable changes in the spectra of MA and MB, even when present in ten equivalent excess. The fluorescence intensity ratios, *F*_403 nm_/*F*_530 nm_ of MA and *F*_403 nm_/*F*_610 nm_ of MB, are shown in Fig. 4b and d. Upon addition of ONOO^–^, they were greatly enhanced by about 4 times and 20 times, respectively, in contrast to other samples. These results indicated that both MA and MB displayed high selectivity toward ONOO^–^ over other common ROS.

**Fig. 5:**
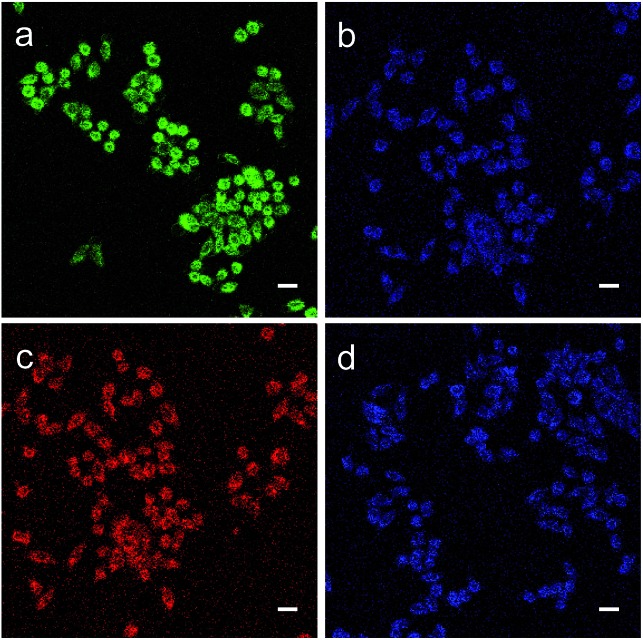
Fluorescence images of HeLa cells for exogenous ONOO^–^. (a and c) The cells were stained with MA (10 mg L^–1^) or MB (10 mg L^–1^) for 1 h and then washed with PBS before imaging; (b and d) the cells were pretreated as (a and c), and then treated with SIN-1(1 mM) for 30 min. (a) In the green channel, (c) in the red channel, and (b and d) in the blue channel. Scale bar: 25 μm.

Subsequently, we evaluated the capabilities of MA and MB for imaging ONOO^–^ in living cells. As shown in Fig. 5a, when the HeLa cells were incubated with MA, they exhibited strong green fluorescence. This suggests that MA is cell-permeable and still has the FRET effect in living cells. By comparison, when the cells were treated with the ONOO^–^ donor SIN-1(3-morpholinosydnonimine), the green fluorescence was quenched and the blue emission from the NMOF was recovered (Fig. 5b), revealing that MA could image exogenous ONOO^–^ in a cellular environment with dual colour switching. A similar FRET phenomenon was observed when the cells were incubated with MB as shown in Fig. 5c and d. The intense red fluorescence resulting from FRET was quenched after treatment with SIN-1, with the recovery of the blue colour. The good cell membrane permeability and interesting dual colour switching of MA and MB enable them to be promising nanosensors for the ratiometric detection of ONOO^–^.

## Methods

### Preparation of a nanoscale metal–organic framework ONOO^–^ sensor

The NMOF was dispersed in DMF (5 mL, 0.5 mg mL^–1^) mixed with 1 mL PVA–ABt or PVA–BDP solution and stirred overnight. The MA or MB product was collected by centrifugation and washed with DMF and water three times to remove excess PVA–ABt or PVA–BDP. The remaining PVA–ABt or PVA–BDP solution and all washing solutions were collected and then diluted to 100.0 mL to measure the absorbency. The concentration of the final PVA–ABt or PVA–BDP solution was calculated using standard curves (Fig. S5†), and the coating amount of PVA–ABt or PVA–BDP on the NMOF was calculated by PVA–ABt or PVA–BDP mass balance in the initial solution and the residual solution.

### General experimental procedure for ONOO^–^ testing by NMOF sensors

All of the photophysical characterization experiments were carried out at room temperature. Absorption spectra were acquired using a Shimadzu UV-3600 spectrophotometer. Fluorescence measurements were performed on a Fluorolog-3 spectrofluorometer, and the sensitivity of the instrument was kept at medium. Deionized water was used to prepare all aqueous solutions. Phosphate buffer saline (PBS, 10 mM, pH 7.4) was purged with nitrogen for 5 minutes before use. MA or MB were dispersed in PBS. ONOO^–^ was prepared by the reported methods.^47^ To test the fluorescence responses of MA or MB towards ONOO^–^ or other reactive agents, MA or MB stock solutions were diluted with PBS and treated with analytes to make sure both MA or MB and the analytes were kept at the desired final concentrations. After quick and vigorous shaking, the mixture was allowed to stand in the dark for the desired time, and the fluorescence spectra were then recorded under an excitation of 340 nm. The emission spectra were scanned from 360 to 650 nm.

### Cell culture and imaging

HeLa cells were kindly provided by the Faculty of Health Sciences, University of Macau. Sterile glass cover slips were put into 6-well plates before the cells were added. The culture solution was a RPMI-1640 medium supplemented with 10% FBS (Fetal Bovine Serum) and 1% penicillin–streptomycin solution. Cells could adhere and grow on the glass cover slips at 37 °C and 5% CO_2_ in a humidified environment. For imaging ONOO^–^ in living cells, cells were incubated with 10 mg L^–1^ MA or MB for 2 h in the RPMI-1640 medium at 37 °C, and then incubated with 1 mM ONOO^–^ donor, SIN-1, for 30 minutes at 37 °C in the same medium. After the incubation, the cells were washed twice with pre-warmed PBS buffer. Fixative solution (Histochoice® Mb Tissue Fixative, Amresco) was added for 15 minutes at room temperature, then a PBS buffer wash was conducted 3 times. Cells without SIN-1 added were also used for imaging in the same process. The samples were observed using a Carl Zeiss Confocal LSM710. All experiments were approved by the institutional committee and performed in compliance with the relevant laws and institutional guidelines of the Faculty of Health Sciences, University of Macau.

## Conclusions

In summary, we have demonstrated a simple yet powerful method for the construction of NMOF based fluorescent probes for the ratiometric sensing of ONOO^–^. PVA is revealed as an effective molecular glue to conjugate with the NMOF surface and arylboronic acid group of molecular probes, yielding energy-transfer nanosensors. The new nanosensors showed a fast response and high selectivity for ONOO^–^ with dual colour switching, which is very suitable for imaging in living cells. We envisage that this approach is generally applicable and should be easily extended to the design of other NMOF based chemical sensors and biosensors.
