# Supplementary material for: Nanoscale metal–organic frameworks coated with poly(vinyl alcohol) for ratiometric peroxynitrite sensing through FRET
Source: Chem Sci. 2017 May 18;8(7):5101–6. doi: 10.1039/c7sc01077j (PMC5613240; doi:10.1039/c7sc01077j)
Supplement: Supplementary file 1 [file SC-008-C7SC01077J-s001.pdf]

Electronic Supplementary Material (ESI) for Chemical Science.
This journal is © The Royal Society of Chemistry 2017

Supporting Information

Nanoscale Metal-Organic Frameworks coated with Polyvinyl Alcohol for Ratiometric Peroxynitrite Sensing through FRET

Zhaoyang Ding,[a, ‡] Jinyun Tan,[a, ‡], Gang Feng,[a] Zhen Yuan,[a] Changfeng Wu,[b] Xuanjun Zhang[*, a]

[a]Faculty of Health Sciences, University of Macau, Macau SAR 999078, China

[b]Department of Biomedical Engineering, Southern University of Science and Technology, Shenzhen, Guangdong 518055, China

*Corresponding author. xuanjunzhang@umac.mo

‡Author contributions. Z. D. and J. T. contributed equally.

Materials

4-(Diethylamino)salicylaldehyde, 4-Bromomethylphenylboronic acid pinacol ester, 2-Benzothiazoleacetonitrile, potassium bifluoride (KHF_2), sodium carbonate (Na_2CO_3), trimethylamine, 4-methylpyridine，4-(dimethylamino)benzaldehyde, piperidine, poly(vinyl alcohol) (Mw 89000-98000), zirconium chloride ($ZrCl_4$) were all purchased from Sigma-Aldrich. All chemicals used in this study were of analytical reagent grade and used without further purification. Ultrapure water (18.25 MΩcm, 25 °C) was used in all experiments.

Synthetic of NMOF

The ligand of NMOF was synthesized by successive Suzuki coupling method according to our previous work[1] as shown in Scheme S1. 20 mg of ligand was dissolved in 10 mL DMF in a glass flask stirring with heated at 120 °C. 5 mL DMF of 3.75 mg $ZrCl_4$ was added into DMF solution drop by drop within 5 minutes. After 10 minutes' reaction, the solution was cooled to room temperature, then the white precipitate was obtained at the bottom of the flask. The product was collected by centrifugation and washed with DMF and ethanol.

Synthetic of PVA-ABt

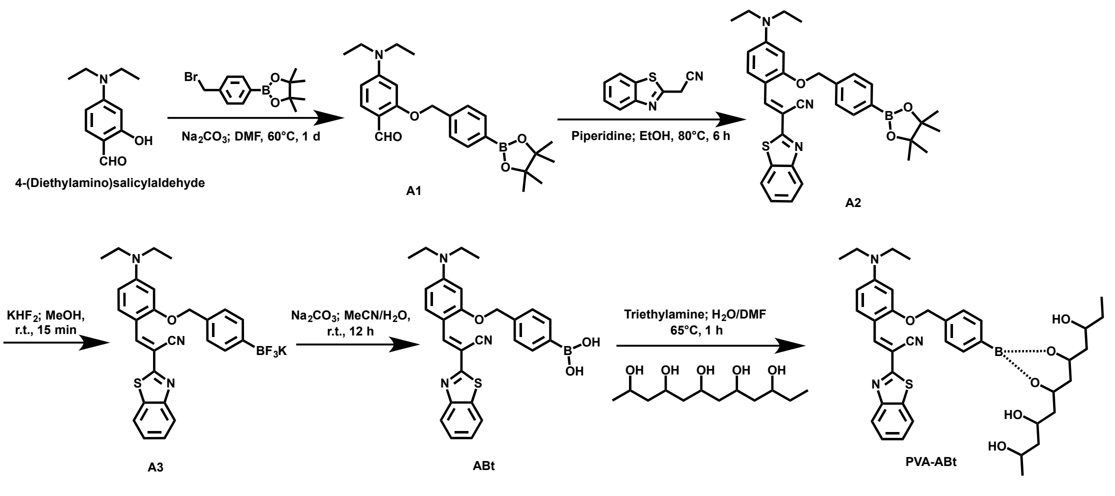

Scheme S1. Synthetic procedure of A1, A2, A3, ABt and PVA-ABt

A1

4-(Diethylamino)salicylaldehyde (1.30 g, 6.7 mmol), 4-Bromomethylphenylboronic acid pinacol ester (2.00 g, 6.7 mmol), and Na_2CO_3 (3.57 g, 33.7 mmol) were added into a 100 mL round-bottom flask containing 40 mL DMF. The mixture was stirred under 60 °C for 1 day. The reaction was cooled to ambient temperature, and diluted with 100 mL distilled water. The mixture was filtered under reduced pressure, and the precipitate

was then recrystallized in methanol to give **A1** as white solid (1.51 g, yield 54.7 %). ^{1}H NMR (400 MHz, Chloroform-*d*) δ 10.26 (s, 1H), 7.85 (d, *J* = 8.1 Hz, 2H), 7.75 (d, *J* = 8.9 Hz, 1H), 7.47 (d, *J* = 8.1 Hz, 3H), 6.30 (d, *J* = 8.9 Hz, 1H), 6.06 (s, 1H), 5.21 (s, 3H), 3.38 (q, *J* = 7.1 Hz, 5H), 1.37 (s, 14H), 1.17 (t, *J* = 7.1 Hz, 8H).

A2[2]

In a 50 mL round-bottom flask, 2-Benzothiazoleacetonitrile (0.47 g, 2.7 mmol) and **A1** (1.00 g, 2.7 mmol) were dissolved in ethanol (30 mL), and then piperidine (100 μL, 1.0 mmol) was added. The mixture was stirred under 80 °C for 4 h. After cooled to ambient temperature, the mixture was filtered under reduced pressure, and the residue was then recrystallized in ethanol to give **A2** as orange solid (0.83 g, yield 60.0 %). ^{1}H NMR (400 MHz, DMSO-*d*$_6$) δ 8.51 (s, 1H), 8.27 (d, *J* = 9.2 Hz, 1H), 8.10 (dd, *J* = 8.1, 1.2 Hz, 1H), 7.98 – 7.94 (m, 1H), 7.77 – 7.74 (m, 2H), 7.56 – 7.53 (m, 2H), 7.51 (dd, *J* = 8.2, 1.2 Hz, 1H), 7.45 – 7.40 (m, 1H), 6.55 (dd, *J* = 9.3, 2.3 Hz, 1H), 6.30 (d, *J* = 2.4 Hz, 1H), 5.38 (s, 2H), 3.46 (q, *J* = 7.0 Hz, 4H), 1.30 (s, 12H), 1.10 (t, *J* = 7.0 Hz, 6H). ^{13}C NMR (101 MHz, DMSO) δ 165.84, 160.58, 153.82, 153.33, 140.75, 140.67, 135.24, 134.04, 129.96, 127.25, 127.00, 125.63, 122.66, 118.75, 109.12, 106.16, 95.55, 94.65, 84.19, 70.14, 44.73, 25.16, 13.00.

A3[3]

In a 50-mL round-bottom flask, **A2** (0.50 g, 0.88 mmol) were dissolved in methanol (20 mL), and then KHF_2 (0.41 g, 5.3 mmol) was added. The reaction was stirred under ambient temperature for 0.5 h. The mixture was concentrated in vacuum and dissolved in hot acetone, and then filtered. The filtrate was concentrated in vacuum, and the residue was recrystallized in mixed solvents of hot acetone and ether, to give A3 as orange-red solid (0.39 g, yield 80.0 %)

ABt

A3 (0.25 g, 0.45 mmol) and Na_2CO_3 (0.24 g, 2.3 mmol) were added into a 25-mL round-bottom flask containing MeCN (10 mL) and distilled water (5 mL). The reaction was stirred under ambient temperature for 1 d. The mixture was acidified with saturated aqueous ammonium chloride (8 mL) and 1 M hydrochloric acid (2 mL), then extracted with ethyl acetate (3 × 10 mL). The combined organic extracts were dried by sodium sulfate, filtered and concentrated in vacuum to give **ABt** as red solid (0.17 g, yield 75.2 %)

PVA-ABt

0.2 g **PVA** was dissolved in 10 mL deionized water and 1.5 mg **ABt** was dissolved in

5 mL DMF, respectively. The solutions were mixed together and triethylamine was used the adjusted the pH of the solution to 8 to 9. The reaction was carried out at 65 °C for 1 h. The standard curve of PVA-ABt concentrations (0.50, 1.0, 2.5, 5.0, 10.0 and 20.0 mg/L) to absorbance at 455.0 nm was determined for further study.

Synthetic of PVA-BDP

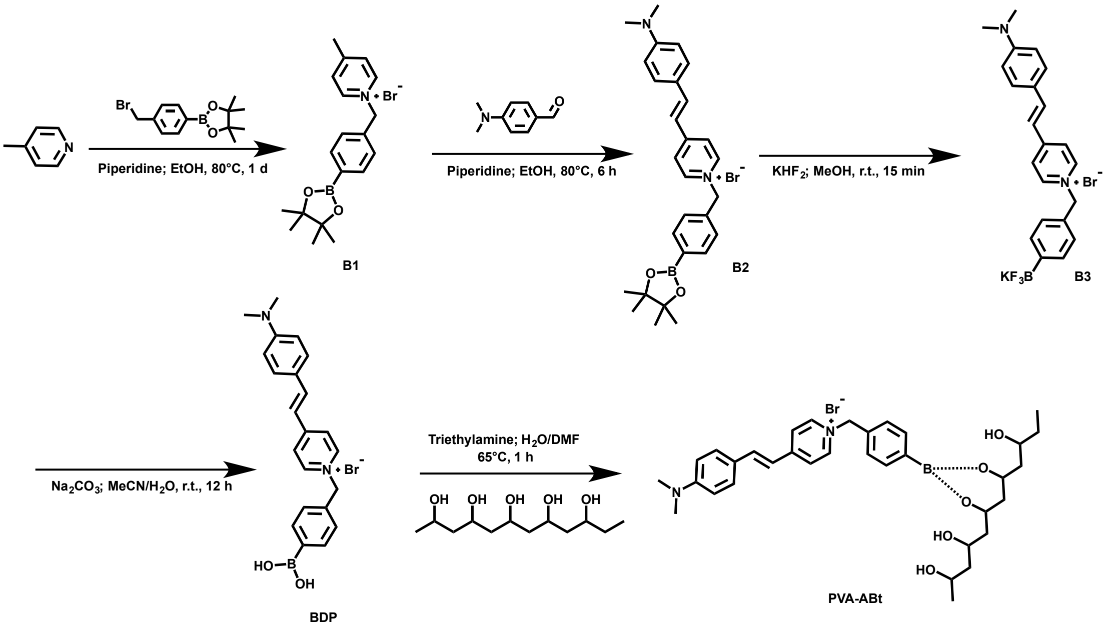

Scheme S2. Synthetic procedure of B1, B2, B3, BDP and PVA-BDP

B1

4-Picoline (0.34 g, 3.7 mmol) and 4-Bromomethylphenylboronic acid pinacol ester (1.00 g, 3.4 mmol) were added into a 50-mL round-bottom flask containing MeCN (30 mL). The reaction was stirred 85 °C for 2 d. The mixture was concentrated in vacuum. The residue was then recrystallized in ethanol/diethyl ether to give **B1** as chalky white solid (1.26 g, yield 90.6 %). ^{1}H NMR (400 MHz, DMSO-d_6) δ 9.03 (d, J = 6.7 Hz, 2H), 8.02 (d, 2H), 7.73 (d, J = 8.1 Hz, 2H), 7.50 (m, 2H), 5.83 (s, 2H), 2.61 (s, 3H), 1.29 (s, 12H). ^{13}C NMR (101 MHz, DMSO) δ 160.04, 144.35, 137.99, 135.62, 129.23, 128.53, 84.37, 62.83, 25.10, 21.94.

B2

In a 50 mL round-bottom flask, 4-(dimethylamino)benzaldehyde (0.29 g, 1.9 mmol) and **B1** (0.50 g, 1.3 mmol) were dissolved in ethanol (30 mL), and then piperidine (100 μL, 1.0 mmol) was added. The mixture was stirred under 80 °C for 6 h. After cooled to ambient temperature, the mixture was concentrated in vacuum. The residual solution was added diethyl ether to precipitate crude product. Recrystallize the crude in ethanol/diethyl ether to give **B2** as deep red solid (0.35 g, yield 53.0 %). ^{1}H NMR (400 MHz, Chloroform-d) δ 8.95 (d, J = 6.6 Hz, 2H), 7.83 (d, J = 7.8 Hz, 2H), 7.79 (d, J = 6.6 Hz, 2H), 7.60 (d, J = 15.8 Hz, 1H), 7.52 (dd, J = 8.3, 5.3 Hz, 4H), 6.83 (d, J = 16.0 Hz, 1H), 6.71 – 6.66 (d, 2H), 5.96 (s, 2H), 3.08 (s, 6H), 1.34 (s, 12H).

^{13}C NMR (101 MHz, $CDCl_3$) δ 154.45, 152.43, 143.32, 136.01, 135.91, 130.79, 128.42, 122.56, 122.30,

116.29, 111.96, 84.10, 62.97, 40.12, 24.85.

B3

In a 50-mL round-bottom flask, **B2** (0.30 g, 0.56 mmol) were dissolved in methanol (20 mL), and then KHF_2 (0.27 g, 3.5 mmol) was added. The reaction was stirred under ambient temperature for 0.5 h. The mixture was concentrated in vacuum and dissolved in massive hot acetone, and then filtered. The filtrate was concentrated in vacuum, and the residue was recrystallized in acetone, to give B3 as deep red solid (0.20 g, yield 68.5 %)

BDP

B3 (0.18 g, 0.36 mmol) and Na_2CO_3 (0.19 g, 1.8 mmol) were added into a 50-mL round-bottom flask containing MeCN (20 mL) and distilled water (10 mL). The reaction was stirred under ambient temperature for 1 d. The mixture was acidified with saturated aqueous ammonium chloride (10 mL) and 1 M hydrochloric acid (2 mL), then extracted with dichloromethane (6 × 20 mL). The combined organic extracts were dried by sodium sulfate, filtered and concentrated in vacuum. The resulting crude was purified by column chromatography on silica gel (dichloromethane/methanol = 20: 1) to give **BDP** as a deep red solid (0.07 g, yield 45.0 %).

PVA-BDP

0.2 g **PVA** was dissolved in 10 mL deionized water and 1.5 mg **BDP** was dissolved in 5 mL DMF, respectively. The solutions were mixed together and triethylamine was used the adjusted the pH of the solution to 8 to 9. The reaction was carried out at 65 °C for 1 h. The standard curve of PVA-BDP concentrations (1.0, 2.0, 5.0, 10.0, 20.0 and 50.0 mg/L) to absorbance at 475.0 nm was determined for further study.

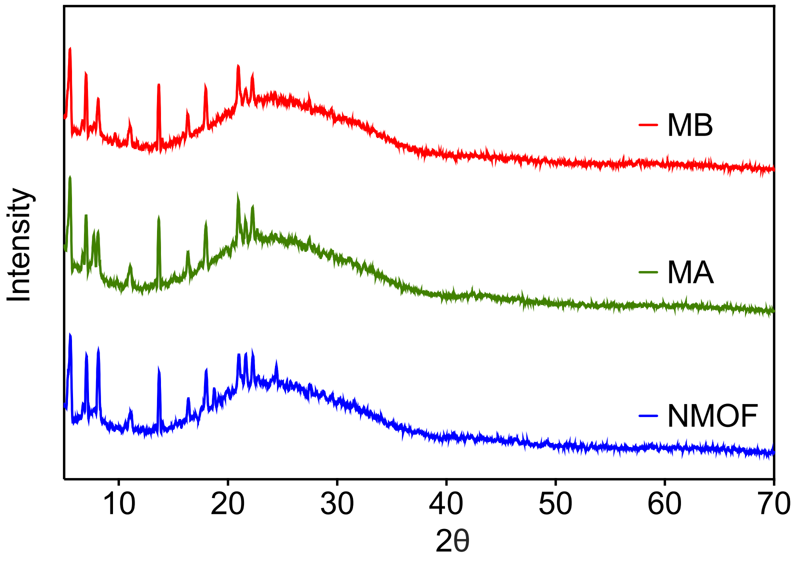

Figure S1. XRD pattern for NMOF, MA and MB

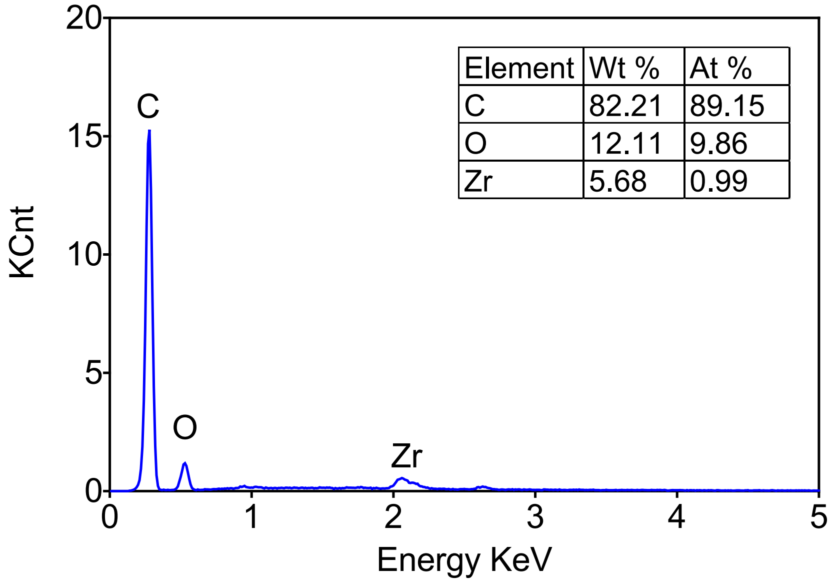

Figure S2. EDS analysis for NMOF

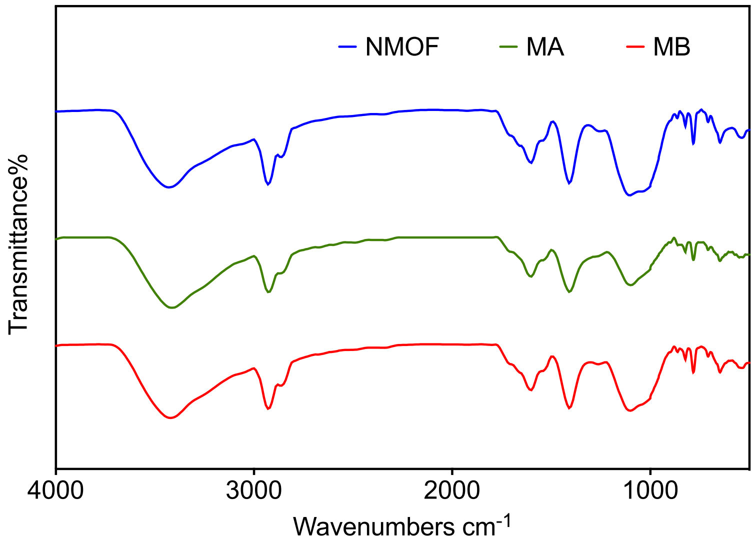

Figure S3. FT-IR spectra for NMOF, MA and MB

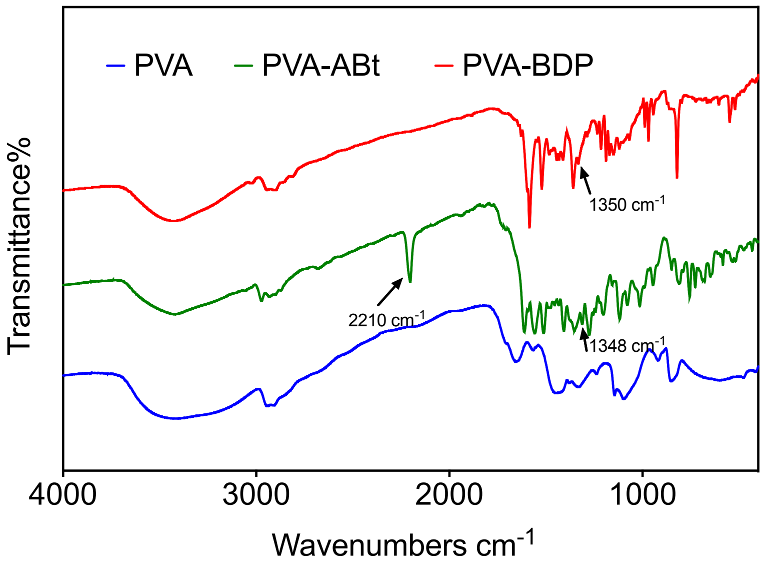

Figure S4. FT-IR spectra for PVA, PVA-ABt and PVA-BDP

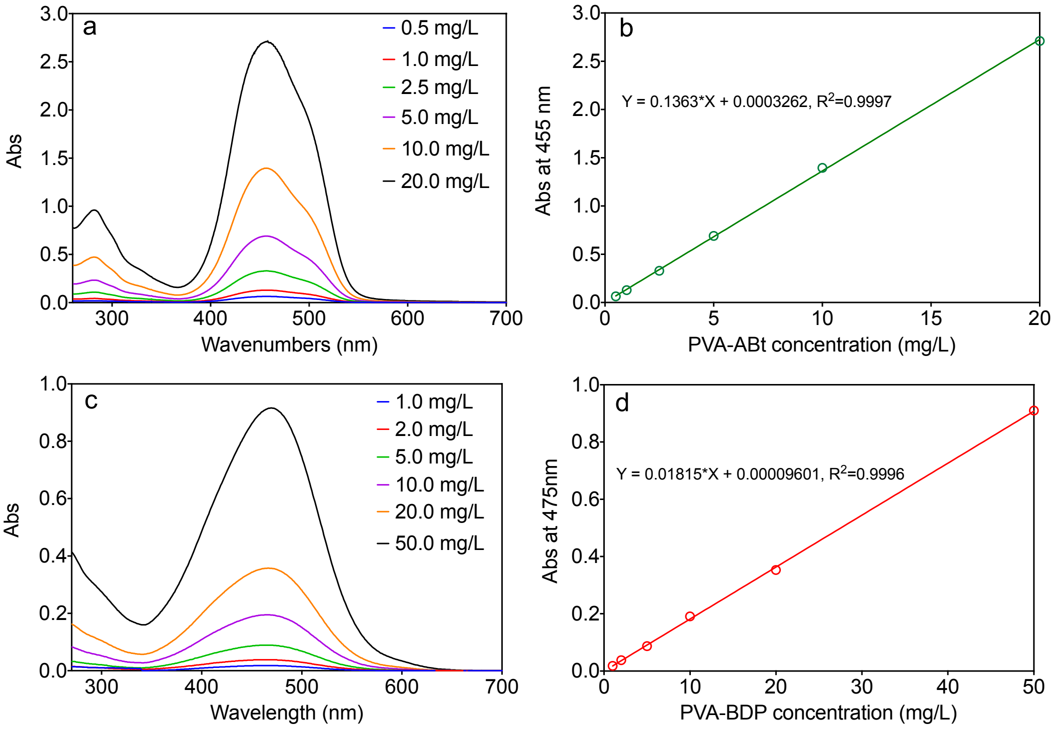

Figure S5. Absorbance spectra of PVA-ABt and PVA-BDP

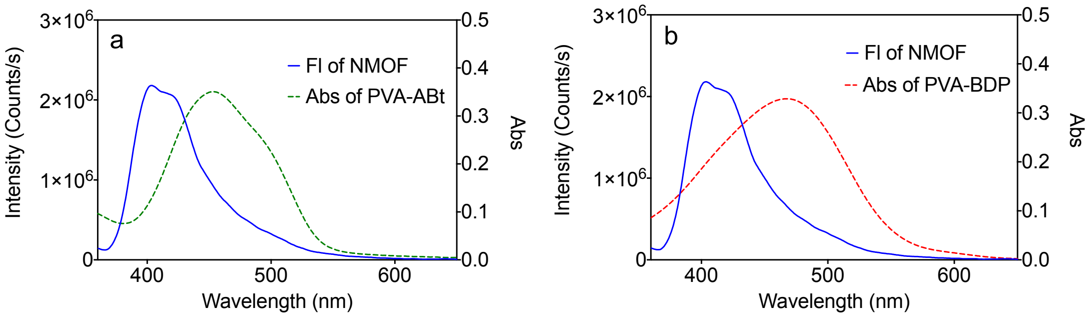

Figure S6. Fluorescence spectra of NMOF and absorbance spectra of PVA-ABt and PVA-BDP

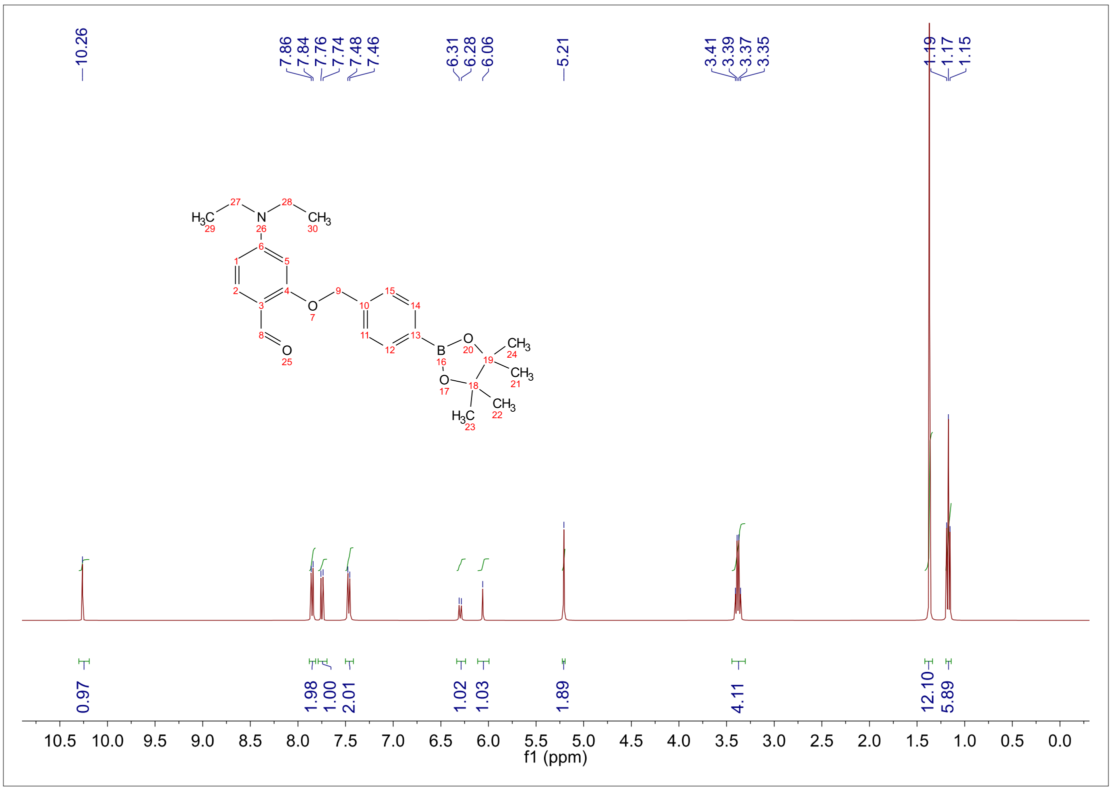

Figure S7. ^{1}**H NMR of A1**

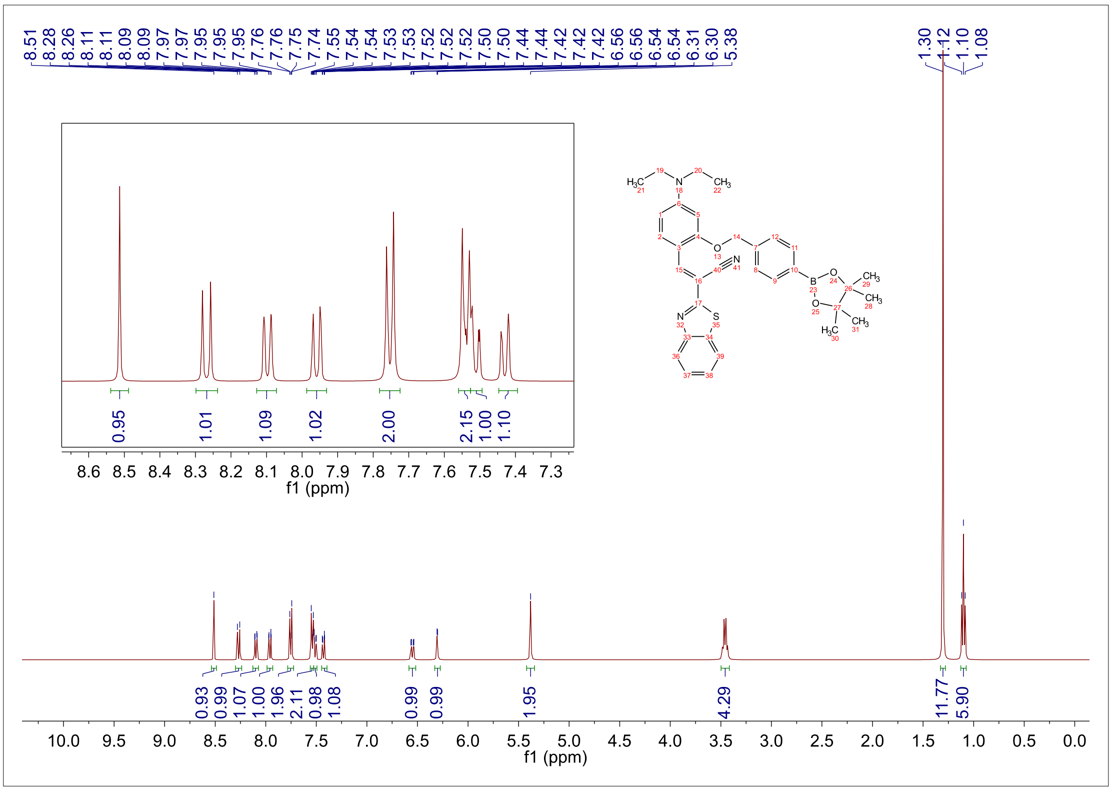

Figure S8. ^{1}**H NMR of A2**

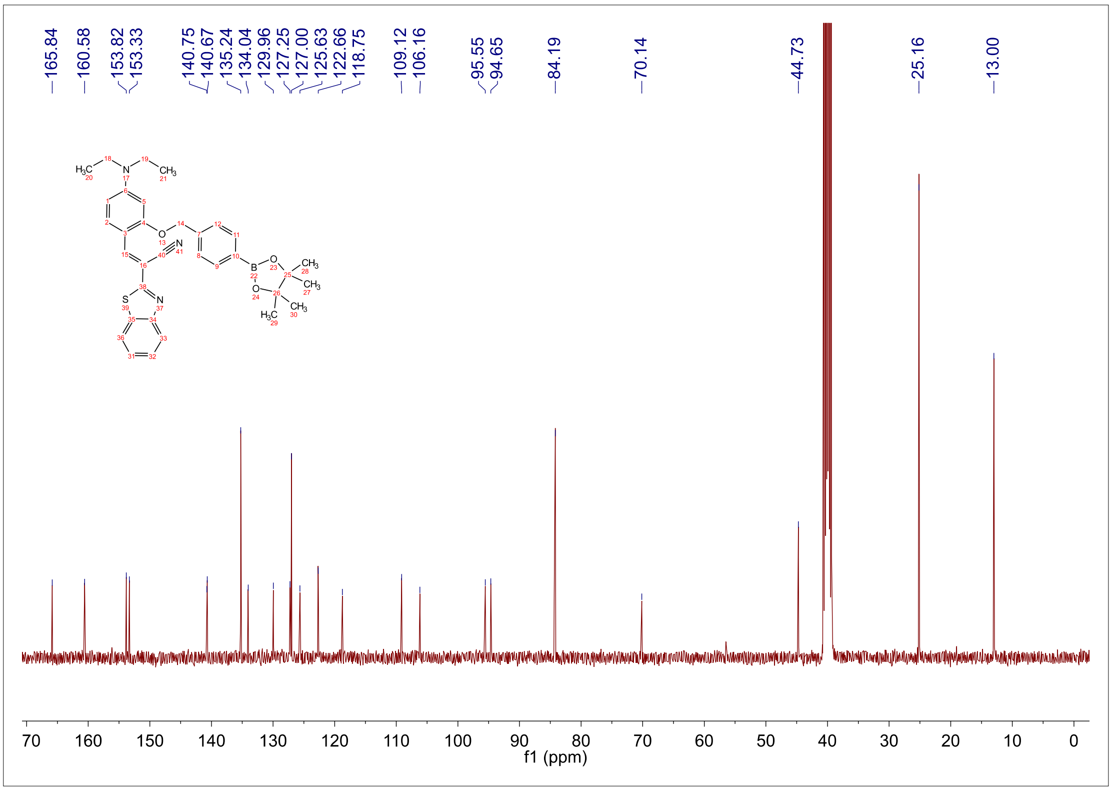

Figure S9. ^{13}C **NMR of A2**

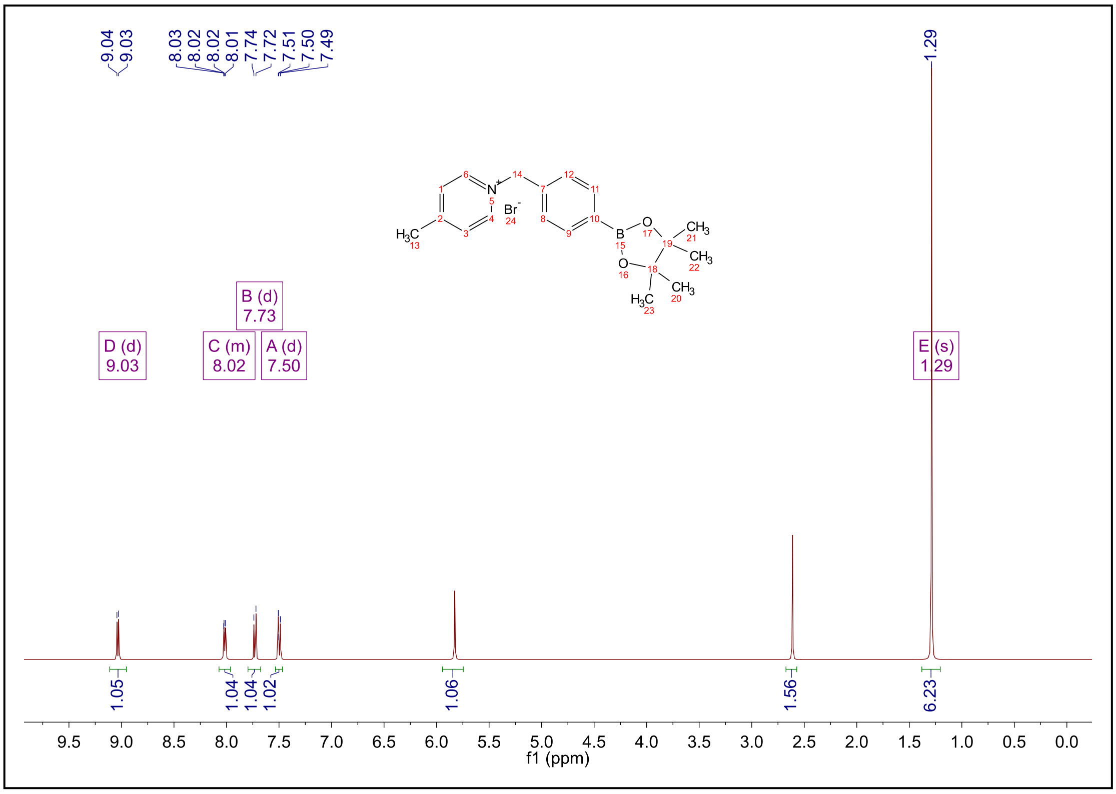

Figure S10. ^{1}H **NMR of B1**

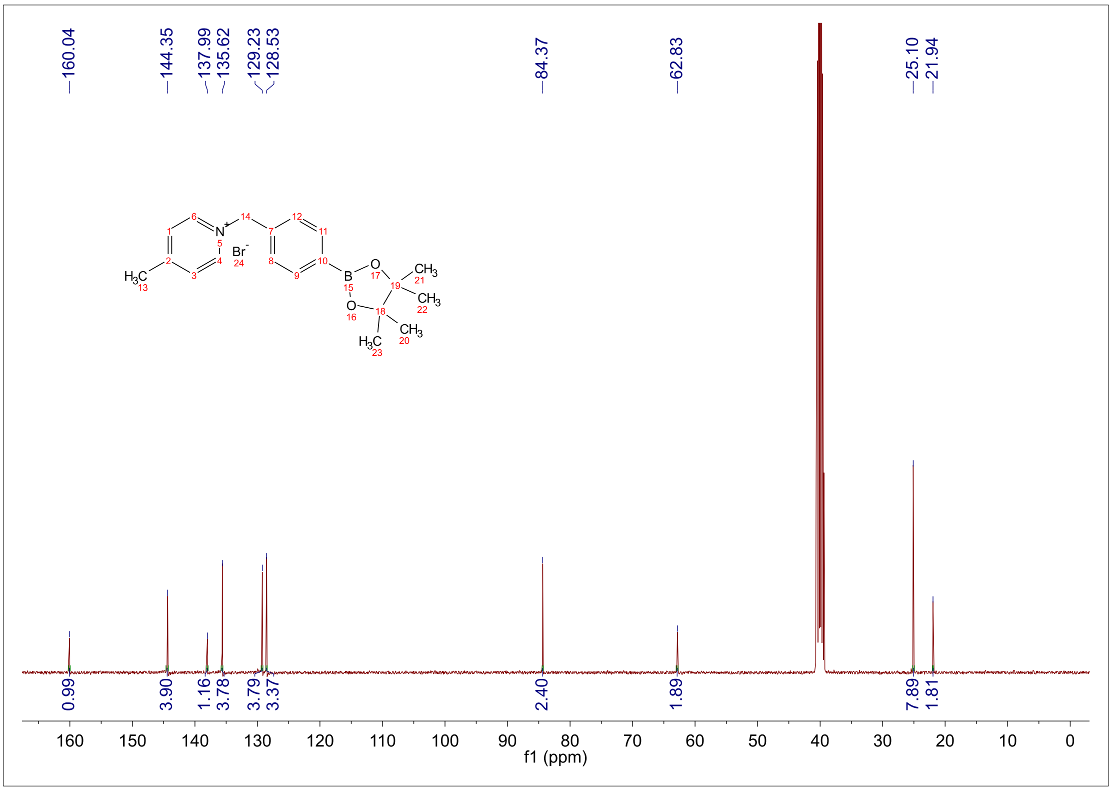

Figure S11. ^{13}C NMR of B1

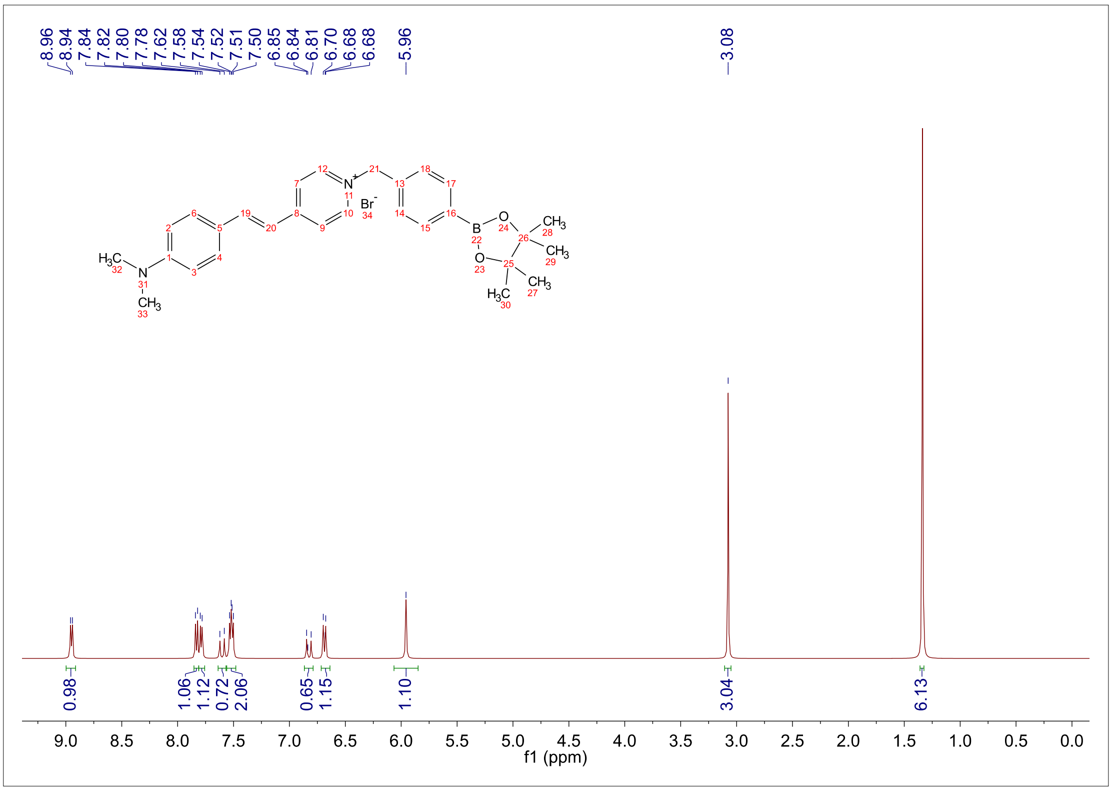

Figure S12. ^{1}H NMR of B2

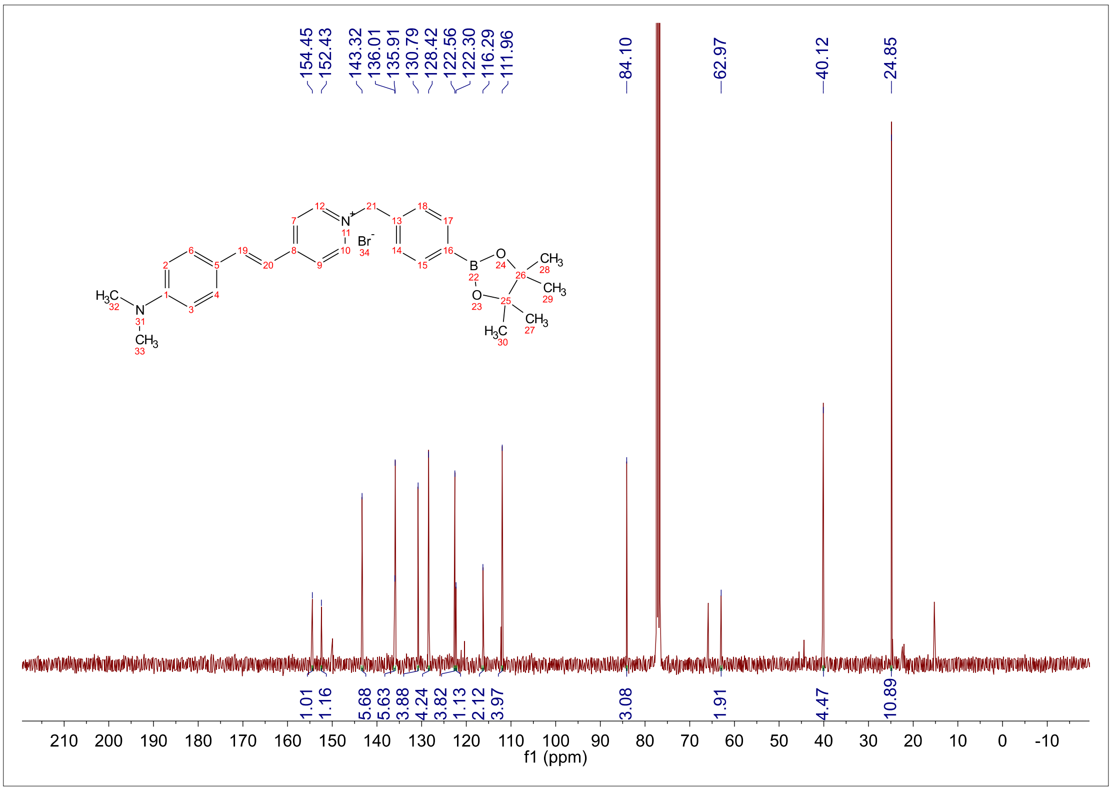

Figure S13. ^{13}C NMR of B2

References.

(1)Zhang, X.; Chen, Z.-K.; Loh, K. P., Coordination-Assisted Assembly of 1-D Nanostructured Light-Harvesting Antenna. *J. Am. Chem. Soc.* **2009,** *131* (21), 7210-7211.

(2)Zhou, J.; Li, Y.; Shen, J.; Li, Q.; Wang, R.; Xu, Y.; Qian, X., A ratiometric fluorescent probe for fast and sensitive detection of peroxynitrite: a boronate ester as the receptor to initiate a cascade reaction. *RSC Adv.* **2014,** *4* (93), 51589-51592.

(3)Yuen, A. K.; Hutton, C. A., Deprotection of pinacolyl boronate esters via hydrolysis of intermediate potassium trifluoroborates. *Tetrahedron Lett.* **2005,** *46* (46), 7899-7903.
